# A Meta-analysis of the Association Between Needle Exchange Programs and HIV Seroconversion Among Injection Drug Users

**DOI:** 10.7759/cureus.3328

**Published:** 2018-09-18

**Authors:** Mohammed U Mir, Faisal Akhtar, Mengxi Zhang, Nicholas J Thomas, Hui Shao

**Affiliations:** 1 Epidemiology and Public Health, Lives and Livelihoods Fund, Jeddah, SAU; 2 Neurology, Ochsner Health System, New Orleans, USA; 3 Epidemiology and Public Health, Tulane University School of Public Health, New Orleans, USA

**Keywords:** needle exchange programs, hiv seroconversion, injection drug users, meta-analysis

## Abstract

We assessed the association between different levels of needle exchange program (NEP) use and human immunodeficiency virus (HIV) seroconversion in the injecting drug user (IDU) population using meta-analysis to aggregate risk estimates from any reported cohort studies or randomized controlled trials (RCTs) in the literature.

We searched the literature for articles published from January 1990 to August 2014 using Medical Subject Headings and other terms from MEDLINE® (using Ovid), Embase, ProQuest, the International Aids Society Abstract database, and the European AIDS clinical society database, and the European Conferences Abstract Archive.

Articles were included if data were reported from an original study; the study was a prospective cohort or RCT design; estimates for seroconversion (hazard ratios [HRs]) for drug-users with different levels of NEP-use, as well as variance data, or the information to calculate these were reported; risk estimates were adjusted for unstable housing, risky sexual behaviors, frequency of injections, cocaine use, and risky needle sharing practices; and the study was published between January 1990 and August 2014. Information abstracted was general study information (i.e., study name, authors, publication year, study site, sample size, length of follow-up, and follow-up intervals, incentives to IDUs for improving compliance/enrollment), outcomes variable measures (seroconversion HR estimates, variance figures, and factors adjusted for), description of the study population (inclusion and exclusion criteria and definitions of comparison groups), type of intervention (NEP program), statistical methods used, and sub-group information.

Two prospective cohort studies with a total of 3,172 IDUs were eligible for inclusion. Comparison groups had different levels of NEP-use (e.g., daily use vs. non-daily use and NEP-users vs. non-users) from fixed-site NEPs. Our Q-statistic was insignificant with a p-value of 0.401 while the I2 value was 0.0%. A random-effects model was used to aggregate the estimates, and we found an overall significant positive association between NEP-use and HIV seroconversion with an HR estimate of 1.59 (95% confidence interval [CI]: 1.2 to 2.1). According to our results, higher usage of NEPs is associated with a higher risk of HIV seroconversion in the IDU population.

The observed association aligns with previous findings of NEP programs being inadequate for HIV control in IDUs. Further research on the topic needs to be done including studies on different NEP designs and how they can be made more effective by combining with other strategies, including the study of IDU characteristics which make them more likely to use safe syringes when they inject.

## Introduction and background

Worldwide, there are nearly 16 million people classified as injecting drug users (IDUs) [1]. The IDU population has high prevalence rates of human immunodeficiency virus (HIV) infections and other blood-borne viruses because of poor injecting risk behaviors (IRBs) such as sharing syringes for drug use. HIV prevalence estimates are as high as 72% (in Estonia), and the prevalence is over 10% in the United States (US), China, and Russia [1]. Besides being at a higher risk of infection themselves, IDUs also pose increased risks of infection for others with whom they are in contact, such as sexual partners.

Needle exchange programs (NEPs) are considered an important strategy for reducing the risk of HIV infection among IDUs and, thus, limiting disease spread. Based on previous effectiveness and feasibility assessments, the World Health Organization (WHO) recommends NEPs for populations threatened by or experiencing an epidemic of HIV among injection drug users [2,3].

Despite the recommendations for NEPs, recent reviews still report inconsistencies in NEP effectiveness [4,5]. The recommendations for NEP adoption are based on reviews which consider positive findings from weaker study designs (mostly cross-sectional and ecological) [3,6,7]. Results from many higher validity studies (cohort designs) have shown either no association or a negative association of NEPs with risks of HIV infection [8-11]. Reviewers have interpreted these negative or insignificant results in terms of bias [12]. However, recent literature indicates previous reviews are more critical of bias introduced in studies with negative findings compared to those reporting positive effects [4].

Furthermore, only a fraction of the studies reported and based their findings on the more objective HIV seroconversion or incidence rates as an outcome measure. Most of the published literature reported changes in IRBs or seroprevalence estimates, which may not necessarily indicate the effectiveness of NEPs in reducing HIV transmission [13]. HIV seroconversion is also a relatively rare outcome deemed by many studies as underpowered (due to insufficient sample size) and potentially not able to detect significant differences [4,6,14].

Our meta-analysis aimed to overcome these gaps in previous reviews. We included studies of high validity with an objective measure of outcome directly representing changes in HIV infection risk (seroconversion) and assessed its association with NEP-use in the global IDU population. Our analysis is also an update of previous reviews as we include more recent cohort studies which tend to have fewer biases and improved study designs [4,15,16].

## Review

Research question

Our primary research question was whether interventional NEP use is associated with HIV infection trends in the IDU population. We assessed the risk of HIV seroconversion among IDUs having different levels of NEP-use (e.g., users vs. non-users, or frequent vs. non-frequent use, etc.) by conducting a meta-analysis of risk estimates (relative risks [RR], hazard ratios [HR], or odds ratios [OR]) from any reported cohort studies or randomized controlled trials (RCTs) in the literature. Additionally, we aimed to assess differential associations in subgroup analyses based on the socio-economic status of the study country and variations in the types of NEP programs.

Eligibility criteria and study selection

Two investigators assessed the eligibility of studies for inclusion independently and in duplicate in all stages of the study selection process. Any discrepancies were resolved through consensus following a discussion. Articles were considered for inclusion in the meta-analysis if the authors reported data from an original study (peer-reviewed and non-peer-reviewed studies were both eligible for inclusion) or the study was a prospective cohort or RCT study consisting of IDUs to ensure higher-level evidence was included in the analysis, thereby improving the validity of our results. The authors must also have reported estimates for seroconversion (including RR, OR or HR) for drug-users with different levels of NEP use, as well as variance data, or the information to calculate these. Due to the rarity of HIV infection/seroconversion, it was considered appropriate to pool the different measures of association. All eligible studies needed to have the RR estimates adjusted for important confounders identified a priori, including unstable housing, risky sexual behaviors, the frequency of injections, cocaine use, and risky needle sharing practices [17-21]. Any studies only reporting unadjusted incidence rates were excluded. The studies had to be published from January 1990 through August 2014. Based on our initial review of the literature, NEP programs existed before 1990, but studies evaluating their effectiveness using seroconversion estimates did not show up until later. We allowed for a broad variety of NEP programs, including standing, mobile, and pharmacy programs to be included in the analysis so we could assess any variations in the association based on the program design. After identifying and removing duplicates, we identified articles eligible for further review by performing an initial screen of identified titles or abstracts. We used the EndNote version 5 (Clarivate Analytics, Philadelphia, PA) reference managing software to identify duplicates and to screen titles and abstracts. Any studies which were eligible for inclusion or could not be rejected due to limited information in the abstract screening were included in the full-text review, after which the final studies were selected. For any articles in which the investigators disagreed on inclusion or exclusion or the reasons for exclusion (based on the eligibility criteria), a final decision was made via discussion with a third investigator. If it was suspected that multiple publications were reporting on the same data, the research team developed a consensus on which results to include. For duplicated or updated results from the same cohort, all else being equal, only the most recent reports were included.

Search strategy

We conducted a systematic search of the literature from January 1990 through August 2014 for studies describing the association of NEP-use with the risk of HIV infection (measured by seroconversion RRs, ORs, or HRs). A comprehensive MEDLINE search was conducted using Ovid, and additional published research was searched using Embase. A search of unpublished theses was performed using ProQuest. We also performed a search of relevant conference proceedings including research abstracts and presentations from the International Aids Society Abstract (IASC) database (2001–2012) and the European AIDS Clinical Society database (EASC), and the European Conferences Abstract Archive (1997–2013). Finally, a bibliography search of all selected articles was used to identify qualifying studies not unearthed by the earlier search strategies. Four search themes were used and combined with the Boolean operator “and”: 1) Needle exchange programs, 2) HIV infection/seroconversion, 3) IDUs, and 4) Cohort or RCTs. The search in the IASC and EASC databases was limited to only two terms for a broader and more comprehensive search as combining the four themes did not retrieve any results. The Medical Subject Headings (exploded versions) and other terms used exclusively to search these databases are shown in Table 1.

**Table 1:** Search terms and syntax used for identifying potentially eligible studies for each database. HIV: Human immunodeficiency virus; RCT: Randomized controlled trial.

OVID Terms:
1. Needle-Exchange Programs/
2. HIV/
3. HIV Seropositivity/
4. HIV Infections/
5. Incidence/
6. Substance Abuse, Intravenous/
7. Needle exchange*.mp. [mp=ti, ab, ot, nm, hw, kf, ps, rs, ui, bt, tx, ct]
8. Needle exchange program*.mp. [mp=ti, ab, ot, nm, hw, kf, ps, rs, ui, bt, tx, ct]
9. Needle syringe program*.mp. [mp=ti, ab, ot, nm, hw, kf, ps, rs, ui, bt, tx, ct]
10. Syringe exchange*.mp. [mp=ti, ab, ot, nm, hw, kf, ps, rs, ui, bt, tx, ct]
11. Syringe exchange program*.mp. [mp=ti, ab, ot, nm, hw, kf, ps, rs, ui, bt, tx, ct]
12. Intravenous drug abuse*.mp. [mp=ti, ab, ot, nm, hw, kf, ps, rs, ui, bt, tx, ct]
13. Intravenous substance abuse*.mp. [mp=ti, ab, ot, nm, hw, kf, ps, rs, ui, bt, tx, ct]
14. Intravenous drug users.mp. [mp=ti, ab, ot, nm, hw, kf, ps, rs, ui, bt, tx, ct]
15. HIV seroconversion.mp. [mp=ti, ab, ot, nm, hw, kf, ps, rs, ui, bt, tx, ct]
16. HIV incidence.mp. [mp=ti, ab, ot, nm, hw, kf, ps, rs, ui, bt, tx, ct]
17. cohort.mp. [mp=ti, ab, ot, nm, hw, kf, ps, rs, ui, bt, tx, ct] 18. Randomized controlled trial.mp. [mp=ti, ab, ot, nm, hw, kf, ps, rs, ui, bt, tx, ct]
Combination of terms with Boolean operators: (1 OR 7 OR 8 OR 9 OR 10 OR 11) AND (2 OR 3 OR 4 OR 5 OR 15 OR 16) AND (6 OR 12 OR 13 OR 14) AND (17 OR 18)
EmBase Terms:
(‘needle exchange program’/exp OR ‘syringe exchange’) AND ‘intravenous drug abuse’/exp AND (‘HIV infection’/exp OR “incidence”/exp OR ‘seroconversion’/exp) [embase]/lim
ProQuest Terms:
‘Needle Exchange Program’ AND ‘HIV’ AND (‘HIV infection’ OR ‘Seroconversion’) AND ‘Intravenous Drug Use’ AND (‘Cohort’ OR ‘RCT’)
International AIDS Society Database Terms:
‘Needle exchange programs’ AND ‘Incidence’
European AIDS Clinical Society Database/European Conferences Abstract Archive:
‘Needle exchange programs’ AND ‘Incidence’

Data abstraction

All data abstraction was completed independently and in duplicate by two investigators. Data abstracted by each reviewer were compared, and discrepancies were resolved by referring back to the abstracts and articles in question. The data abstraction form included general study information (e.g., study name, authors, publication year, study site, sample size, length of follow-up and follow-up intervals, and incentives to IDUs for improving compliance/enrollment), adjusted outcomes variable measures (seroconversion HR estimates, variance figures, and factors adjusted for), description of the study population (inclusion and exclusion criteria and definitions of comparison groups), type of intervention (NEP program), statistical methods used, and sub-group information.

Assessment of study quality

Study bias was assessed at both the design and analysis phases. To include only high-quality studies, the selection of publications was limited to cohorts and RCTs. We assessed the quality of studies by looking at recruitment procedures, method of group assignment, losses to follow-up, adequate follow-up periods, confounders which they adjusted for, statistical methods, and use of blinding (if any). We did not develop a quality scoring mechanism in our analysis as the practice has been found to be controversial [22,23].

Summary measures and synthesis of the results

HR estimates were used to examine the association between the NEPs and HIV/AIDS seroconversion. Any heterogeneity of program effects across studies was evaluated by the Cochrane Q statistic (p < 0.10 was used as a threshold to represent statistically significant heterogeneity) and the I2 statistic (values of 25%, 50%, and 75% represent low, medium, and high heterogeneity, respectively) [24,25]. We ran both fixed and random effect models for the analysis but present results only from the random effects as it provides a more conservative estimate of the effect size given the highly underpowered tests for heterogeneity (Q-statistic) due to the low number of studies available for inclusion in the analysis (despite assigning a higher level of significance [p < 0.1]). The inverse variances of the reported estimates were used to weigh the results. Forest plots were visually assessed for the HRs with their corresponding 95% confidence intervals (CI) across studies. The analysis was performed using the statistical software package STATA version 12.0 (StataCorp, College Station, TX).

Our a priori analysis plan outlined further sub-group analysis (based on study country income, NEP design, and study design), sensitivity analysis (based on the quality assessment of studies), and assessment of publication bias (using visual inspection of funnel plots with standard errors plotted against effect size, the Egger regression test [26], and the trim and fill method to detect “missing” studies in the funnel plot and to simulate results by their inclusion) [27]. However, we could not proceed with these due to a limited number of eligible studies resulting from our search. We describe the implications of this limitation further in the discussion section.

Literature search

**Figure 1:**
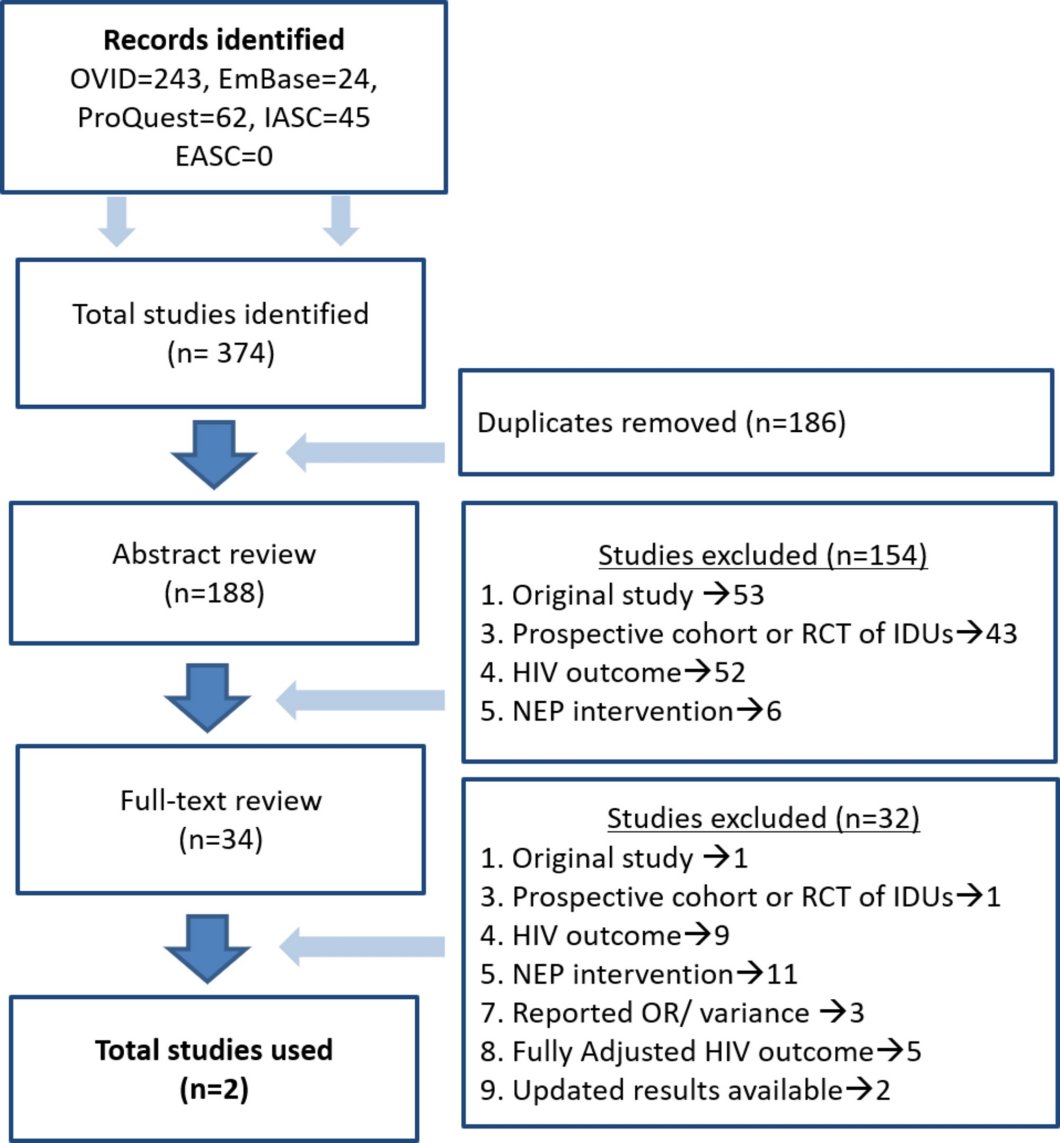
Literature search. IASC: International Aids Society Abstract database; EASC: European AIDS Clinical Society database; IDU: Injection drug user; RCT: Randomized controlled trial; HIV: Human immunodeficiency virus; NEP: Needle exchange program; OR: Odds ratio.

Our initial search of the databases using the search terms returned 374 potentially eligible studies. After removing duplicates (n = 186), 188 articles remained for title and abstract screening. A majority of these (n = 154) were excluded at this stage. The reasons for exclusion, based on our eligibility criteria, are shown in Figure 1. The full-text review of 34 studies led to the exclusion of a further 32, with two studies remaining [16,28] which were included in the analysis. No eligible RCTs were found for inclusion. In the screening and review process, RCTs were primarily excluded because of ineligible outcome measures. None of the RCTs looked at the effect of NEP-usage on the risk of HIV seroconversion and were mostly focused on changes in the IRBs in IDUs attending NEPs.

Study characteristics

The selected study characteristics are shown in Table 2. Both Bruneau et al. [28] and Wood et al. [16] are prospective cohort designs with Canadian IDU populations (open cohorts from Montreal and Vancouver, respectively). Wood et al. include participants from the Vancouver Injection Drug Users Study which enrolls through street outreach while Bruneau et al. recruited the St. Luc Cohort from outreach activities and community programs. Both are relatively recent studies with publication dates in the late 2000s. Information on 3,172 participants from both studies on NEP-use behavior and the risk of HIV seroconversion was included in our analysis. A total of 281 IDUs seroconverted during the 48-month follow-up period for both studies. IDUs who did not return for at least one follow-up visit were likely to be younger and more frequent drug injectors compared to those who did. All participants were HIV-negative at the time of enrollment. The NEP programs in both studies were similar in design and dispensed as many as required sterile syringes to IDU attendees from fixed-sites [16,28].

**Table 2:** Characteristics of studies included in the meta-analysis. HIV: Human immunodeficiency virus; NEP: Needle exchange program.

	Bruneau et al. (2011) [28]	Wood et al. (2007) [16]
Study Site	Montreal, Canada	Vancouver, Canada
Study Design	Prospective cohort study	Prospective cohort study
Number of participants	2137	1035
Recruitment criteria	18 years and above, having injected drugs in last six months, at least one follow-up visit, HIV-negative	Injected at least once in last month, resident of greater Vancouver area, at least one follow-up visit, HIV-negative
Recruitment procedures	Voluntary direct street-level, word-of-mouth referral, or community programs	Self-referral, street outreach
Recruitment periods	1992-2001, 2004-2008	1996-2004
Follow-up period	48 months or until seroconversion	48 months or until seroconversion
Follow-up intervals	Three months (first follow-up), then six months	Six months
Comparison groups (NEP-use)	Users vs. non-users	Daily users vs. non-daily users
Number of HIV Seroconversions	148	133
Type of NEP program	Fixed-site	Fixed site

IRBs and other risk behaviors were assessed by bi-annual interviews conducted at each follow-up visit eliciting self-reported information on behaviors practiced in the prior six months. HIV seroconversion was assessed using HIV-1 antibody-enzyme immunoassays with venous blood samples taken at every follow-up visit. Both studies provided comparable monetary compensation to participants in the visits.

The definition of comparison groups was different with respect to the intervention with Wood et al. comparing the risk of seroconversion between daily users of NEPs and non-daily users while Bruneau et al. compared risk estimates of NEP-users with non-users. Baseline characteristics for comparison groups were significantly different in both studies as well, with Wood et al. reporting daily users being more likely to have unstable housing and to be involved in sex trade, to have more frequent heroin and cocaine use, and were more likely to inject at shooting galleries (based on six-month behavior prior to enrollment in the study) [16].

The design and conduct of the studies were comparable in terms of quality. Recruitment, assessment exposures and outcome, and statistical methods used were similar. Both HR estimates were adjusted for IRBs, risky sexual behaviors, the frequency of injecting, and for cocaine use. Adjustment was also done for basic demographics (e.g., age and sex) and unstable housing. Wood et al. additionally adjusted for ethnicity, methadone use, and for geographical proximity of housing to the central needle exchange program in Vancouver. The person-years of observation were adequate for detection of a rare outcome such as HIV seroconversion. However, the number of observed person-years was much higher for Bruneau et al. [16,28].

NEP use and risk of HIV seroconversion

**Figure 2:**
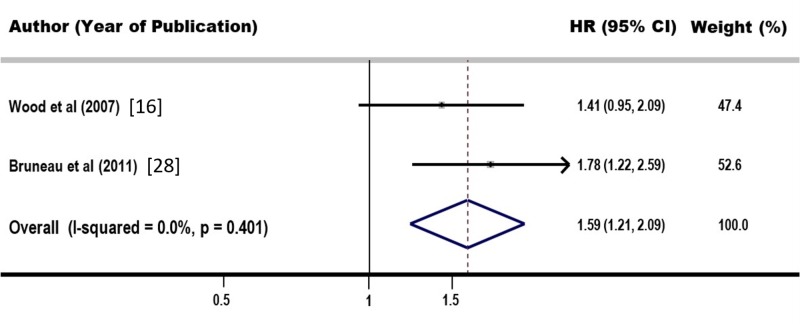
Funnel plot showing adjusted hazard ratios comparing higher vs. lower needle exchange program-use. CI: Confidence interval; HR: Hazard ratio.

Both adjusted HRs showed a positive association between NEP-use and risk of seroconversion, but the estimate from Wood et al. was statistically insignificant. Our Q-statistic was insignificant with a p-value of 0.401 while the I2 value was 0.0%. This, however, was not considered an indication of no-heterogeneity but rather a result of highly underpowered assessment tests. A random-effects model was thus used to aggregate the estimates for a more conservative final estimate. Results of the random effect model are illustrated in Figure 2. We found an overall significant positive association between NEP-use and HIV seroconversion with an HR estimate of 1.59 (95% CI: 1.2 to 2.1) using the model. According to our model, higher usage of NEPs is associated with a higher risk of HIV seroconversion in the IDU population.

Previous literature on this topic has reported conflicting results. As mentioned before, one of the primary reasons for conducting this meta-analysis was to try to establish aggregated, more valid estimates of the association between NEPs and HIV seroconversion. Our results align with previous prospective studies done on the subject which have shown insignificant or negative impacts of NEP programs on HIV infection [8-11,15]. Our literature review revealed only one previous meta-analysis on the subject [29], which included information on three IDU cohorts from the Greater New York City metropolitan area. Des Jarlais et al. [29] report a significant protective effect of NEP programs against HIV seroconversion (non-users vs. continuing users HR of 3.5 [95% CI: 1.3 to 9.7]). However, several possible explanations may account for the different findings. IDUs included in this previous meta-analysis were from three separate IDU projects in New York City. One cohort was of registrants of an NEP program, another was from a methadone treatment study consisting of both NEP-users and non-users, while the third cohort was selected from the very-high-seroprevalence National AIDS Demonstration Research sites which was consisted of non-users of NEPs due to a lack of access. The possibility of selection bias cannot be ruled out here—those selected as NEP-users may potentially have different health-related characteristics compared to the non-users. This could have been enhanced further by the highly restrictive selection criteria where NEP-users were those who reported usage on every follow-up visit while non-users were defined as those who never reported any use on any visit.

Furthermore, important confounders such as risky sexual behaviors, IRBs, cocaine use, and methadone use (particularly relevant due to the source of recruitment) were not adjusted for in the final estimates. Also, the person-years of observation were much less with our analysis having twice the number of participants. The difference can be explained by the authors’ description of the study population as well where they state that users of the NEPs used these programs exclusively and obtained all syringes used from these sites. These differences greatly limit the generalizability of Des Jarlais’s findings and can potentially account for the different results from our analysis.

The exclusive use of sterile syringes by IDUs ensures a reduced risk of HIV seroconversion. A separate regression model run by Bruneau et al. looking at the risk of seroconversion in IDUs who obtained 100% of their syringes from safe sources is less likely to seroconvert compared to those who do not [28]. Wood et al. give a similar explanation of the negative impact of NEP-use in their study attributing the higher risk levels of seroconversion to other risk behaviors of the attendees [16]. This, however, raises a question about the effectiveness of NEP programs in general. These NEPs are among the highest ranked in North America, providing access to sterile syringes to IDUs [14]. Counseling on HIV risk behaviors was also a part of the intervention with IDUs counseled at every follow-up visit. Still, NEP use is correlated with higher seroconversion rates even after controlling for several important confounders. However, there is a possibility of differential misclassification of HIV risk behaviors due to the self-reported information gathered from the IDUs. Self-reported risk behaviors are not always reflective of actual behaviors in IDUs, and other studies have shown that inaccuracy increases with rising frequency of target behaviors [30-32]. If HIV risk behaviors are positively associated with more frequent injecting (and thus, higher NEP-use) and if individuals with high-risk behaviors are less likely to report them, we would see a positive association between NEP use and seroconversion, even though the cause may be other risk behaviors. This would lead to unadjusted residual confounding by HIV risk behaviors, which would distort the association estimates.

Besides using self-reported information for risk behavior assessment, other design issues need to be considered while interpreting the results of the studies included. Participants who could not be included in the analyses were younger and more frequent IDUs in general. The study populations were, thus, different from the general IDU populations in these study sites with these characteristics and potentially on other characteristics not measured. Significant baseline differences existed between the comparison groups in both cases. The observed differences were adjusted for in the analysis, but any unmeasured differences (potentially associated with HIV seroconversions such as exposure to other control interventions and social network characteristics) would still have remained and influenced the results due to the observational nature of the studies.

A major limitation of our meta-analysis is the low number of eligible studies identified in the search. We were not able to assess any heterogeneity in the study results as the Q-statistic was highly underpowered. We compensated this to some extent by using a random effects model to account for both within-study and between-study variation that may exist to pool the study results. Publication bias could also not be assessed. A meaningful funnel plot cannot be obtained using only two studies, and we did not consider an Egger regression test appropriate in the situation either. We did, however, take every measure to limit any publication bias in the design of the meta-analysis. Unpublished literature sources such as dissertations, conference proceedings, and abstracts were searched for any eligible studies, and we did not limit our search to only English language publications as it has been documented that publication bias is higher in English journals [33]. The literature screening and data abstraction was done by two investigators independently and in duplicate with any discrepancies resolved by discussion and referral to original articles, thereby minimizing the likelihood of any selection bias for inclusion. We were unable to carry out our a priori plan of sub-groups and sensitivity analysis among the eligible studies. Both studies included are from the developed world (Canada) and have fixed-site NEPs with freely accessible sterile syringes for exchange. These program designs and other general socio-economic indicators are different from those in other sites, especially in developing countries, thereby limiting the generalizability of our findings.

## Conclusions

Our study results highlight the importance of a comprehensive approach to HIV control for the IDU population that includes exclusive use of sterile syringes via NEPs in addition to other interventions such as counseling and education. More evidence is required for definite conclusions on the effectiveness of NEPs. RCTs on the subject are unethical so further higher quality observational studies looking at the association between NEP usage and HIV seroconversion must be conducted. These studies need to look at the different NEP designs implemented at all locations where HIV is on the rise in IDU populations. It is also essential to study IDU characteristics which are associated with obtaining needles from safe sites and the effect of combined interventions where NEPs are implemented along with other strategies for HIV prevention. This information is critical for enhancing NEP usage and effectiveness to ultimately contribute to IDU well-being.
